# Supplementary material for: Are carbon emissions trading and green financial instruments synergistic? -Comprehensive quantitative research based on content analysis
Source: PLoS One. 2024 Mar 7;19(3):e0298601. doi: 10.1371/journal.pone.0298601 (PMC10919601; doi:10.1371/journal.pone.0298601)
Supplement: S1 Data — (ZIP) [file pone.0298601.s001.zip › Data/地级市Z_F工作报告.zip!/╜¡╬≈/╕╙╓▌╩╨/└┤╫╘╣1⁄2╓┌║┼í╛┬φ┐╦╩2╛▌═°í┐_2015.docx]

各位代表：

现在，我代表市人民政府，向大会作政府工作报告，请予审议，并请各位政协委员和列席会议的同志提出意见。

一、2014年工作回顾

过去一年，在市委的坚强领导和市人大、市政协的监督支持下，全市上下深入贯彻落实党的十八大、十八届三中、四中全会和习近平总书记系列重要讲话精神，坚持发展为先、生态为重、创新为魂、民生为本，全力以赴稳增长、促改革、调结构、惠民生，加快建设创业、宜居、平安、生态、幸福赣州，经济社会发展稳中加速、提质增效，振兴发展取得阶段性重大成效。

——经济实力明显增强。主要经济指标增幅高于全国、全省平均水平，部分指标居全省前列。实现地区生产总值1843.6亿元，增长10%，总量全省第二，增速全省第三。固定资产投资1608.8亿元，增长20.9%，增速连续三年居全省首位。财政总收入328.5亿元，增长17.2%，一般公共预算收入225.3亿元，增长22.2%，增幅均居全省第一，提前一年实现“十二五”规划目标。县域经济实力提升，县（市、区）财政总收入均超6亿元，章贡区、赣州经济技术开发区超30亿元，11个县（区）财政总收入在全省排位前移，十分难得的是安远、寻乌、石城三县均比上年前移1位。

——产业升级明显加快。规模以上工业主营业务收入突破3000亿元，达3002.8亿元，增长14.9%。稀土钨产业集群主营业务收入率先突破千亿元，家具产业集群突破700亿元，主营业务收入超300亿元的产业集群达到5个。战略性新兴产业主营业务收入达400亿元，增长20%。赣南脐橙以53.89亿元的品牌价值居全国农产品价值榜榜首。

——科技创新明显进步。赣州高新技术产业园区以升促建稳步推进，国家离子型稀土资源高效开发利用工程技术研究中心、脐橙工程技术研究中心建设进展顺利，赣州国家钨和稀土新材料高新技术产业化基地获批，国家家具产品质量监督检验中心建成运营，青峰药业创国家技术创新示范企业。“弱磁性矿石高效强磁选关键技术及装备”项目获国家科技进步二等奖。新增高新技术企业6家，总数达63家。专利申请量、授权量均居全省第二。

——基础设施明显改善。昌赣客专正式开工，将结束赣南无高铁的历史。赣韶铁路通车运营。高速公路通车里程突破1000公里，占全省近1/4，实现县县通高速。黄金机场改扩建稳步推进。赣州南500千伏等25项输变电工程投入运营，实现县县至少有2座110千伏变电站。

——平台支撑能力明显提升。全省首个综合保税区——赣州综合保税区加快建设。全国内陆首个进境木材国检监管区——赣州进境木材监管区和定南公路口岸作业区建成运营，进境木材和脐橙出口实现集装箱运输全直通。赣州、龙南、瑞金经济技术开发区和赣南承接产业转移示范区、瑞兴于经济振兴试验区等重大平台建设顺利推进，为发展升级提供了强大支撑。

——人民生活水平明显提高。全市民生类支出303.1亿元，增长11.6%，占财政总支出比重达56.6%，连续三年超过55%。城镇居民人均可支配收入22935元，增长10.3%，增速全省第二；农民人均可支配收入6946元，增长11.6%，增速连续两年全省第一。农村危旧土坯房改造基本完成，规划内农村饮水安全问题全面解决。减少贫困人口34.4万人，四年累计脱贫近110万人。

一年来，面对复杂严峻的国内外发展环境和经济下行压力，在以文清同志为班长的市委带领下，全市上下沉着应对，主动作为，迎难而上，及时采取了一系列有力举措。重点做了五个方面的工作。

（一）全力以赴落实《若干意见》，振兴发展迈出坚实步伐。积极推动政策、项目、资金、平台加速落地。《赣闽粤原中央苏区振兴发展规划》获批实施，国务院及国务院办公厅印发支持文件增至10个，部委支持文件增至89个，整体性、系统化的政策支持体系加速形成。第二次部际联席会议和省领导小组第二、第三次会议相继召开，国家、省、市三级联动推进机制进一步完善。全面执行西部大开发各项优惠政策普遍落实。争取37个部委出台46个对口支援具体实施意见或支持政策，明确或落实到位的援助项目150个、援助资金42亿元。全年争取中央和省级财政下拨各类补助资金359.1亿元。加快重大项目建设，169个市属重点工程完成年度投资619亿元。宁都至定南高速公路开工建设，南昌至宁都、兴国至赣县高速公路建设加快推进，寻全高速安远至信丰段建成通车。

（二）全面深化改革开放，发展活力持续迸发。争取国家层面批复试点示范事项6个，总数达34个。部省共建赣州教育改革发展试验区、全国集体林权制度改革试验示范区建设等取得初步成效。出台全面深化改革配套文件21个。完成农村集体土地所有权确权登记。国有林场改革基本完成。在全省率先开展农民住房财产权抵押贷款试点。工商登记制度改革成效明显，新登记的私营企业及其注册资本分别增长57.8%和80%。大力推进金融改革创新。全省首家网络小额贷款公司、首家商业保理外资金融服务机构开业运营，组建赣南金融资产交易中心、赣州市金融研究院，设立前海股权交易中心赣州办事处，3家企业在“新三板”挂牌上市，4家农信社成功改制为农商行，全市各类金融机构达158家，赣州成为周边四省九市金融机构聚集度领先、门类最为齐全的城市。财政预算、国资国企、生态文明、文化体制、社会治理、瑞金省直管县等改革稳步推进。扎实开展“招商引资项目落地年”活动。全市实际利用外资12.2亿美元，增长10.4%；引进省外5000万元以上项目资金524.2亿元，增长15.4%。外贸进出口总额39亿美元，增长18.3%。其中出口总额32亿美元，增长9.8%，增速提高7.2个百分点。

（三）做大做强特色产业，产业结构加速优化。出台做大做强工业主导产业、加大小微企业帮扶力度等政策措施，着力解决企业融资难、用工难等问题。“财园信贷通”“小微信贷通”发放贷款48.2亿元，“财政惠农信贷通”发放贷款5.2亿元。大力实施优势矿产业高端化、传统产业转型、战略性新兴产业培植工程。赣州稀土集团牵头组建中国南方稀土集团获国务院批准。玖发新能源汽车11款车型进入国家推广目录。成功举办首届中国（赣州）家具产业博览会，家具产业影响力明显提升。规模以上工业增加值751.9亿元，增长12.4%，增速列全省第三，比上年前移1位。

工业用电量85亿千瓦时，增长11.2%，增速列全省第三，比上年前移2位。提升发展优势特色农业。粮食总产实现“十一连丰”。成功举办赣南脐橙网络博览会，通过网上销售带动线下销售，果农收入增长33%。柑橘黄龙病防控扎实有效。生猪、蔬菜、油茶、茶叶等产业持续发展。信丰县成功申报创建国家现代农业示范区。加快区域性金融、物流、旅游中心建设。金融机构存款、贷款余额分别增长9.8%和20.8%，存贷比达66.8%，提高6.1个百分点；金融业税收23.6亿元，增长44.3%。旅游接待总人数3502.8万人次，增长35.2%；旅游总收入279.6亿元，增长35.2%。物流业增加值135.4亿元，增长12.6%。获批创建国家电子商务示范城市，电子商务交易额117.5亿元，增长230%。服务业增加值占比提高0.4个百分点。

（四）加快区域协调发展，城乡面貌深刻变化。以南康撤市设区为契机，加快赣县、上犹、崇义与中心城区同城化。中心城区建成区面积扩大到138.6平方公里、城市人口达138.2万人，“小马拉大车”格局明显改变。教育网点、给水排水等16项专项规划加快编制。“四桥九路”等重大城建项目顺利推进，沙石大桥竣工通车。郁孤台历史文化街区修缮保护工程基本完工。上犹江饮水工程前期工作扎实推进。新建、改造体育场馆7个，新（改）建公厕20座，章江南岸截污干管工程和污水处理厂二期工程竣工。龙南、瑞金两个市域副中心城市加快建设。县（市、区）在建城建项目1450个，增长27.6%。17个示范镇实施项目431个，完成投资12.5亿元。27个镇被列为全国重点镇。推进1744个和谐秀美乡村点建设，建成317个新型农村社区。大力开展“净空、净水、净土”行动。节能减排和淘汰落后产能完成省下达任务。完成造林61.5万亩。赣江源、东江源生态保护得到加强。陡水湖列入全国水质较好湖泊生态环境保护总体规划。空气质量优良率、城镇集中式饮用水水源地水质达标率均保持100%。荣膺全国首批创建生态文明典范城市、全省首届生态宜居城市。

（五）努力增进民生福祉，发展成果普惠于民。始终坚持把改善民生作为振兴发展的首要任务，加快解决突出民生问题。省下达的50件民生实事全部完成，我市安排的40件民生实事当年任务基本完成。农村危旧土坯房改造完成14.28万户，累计改造63.08万户，263万农民告别透风漏雨的土坯房。基本建成保障性住房1.96万套，完成城市棚户区改造安置1.56万户。建设章江新区农民返迁安置房126.2万平方米，回购70.1万平方米。完成40.5万农村人口饮水安全工程，累计解决278.4万农村人口饮水安全问题。农网改造升级新完成低电压治理6.28万户。新建改造农村公路3800公里，三年累计7900公里。市中心城区全面实行公交1元票价制，新投放新型环保公交车150台，开通至龙南、瑞金、上犹城际快线。城镇新增就业9.2万人，新增转移农村劳动力15.7万人。城乡低保、城镇“三无”特困群众和农村五保供养对象保障标准稳步提高，新增农村低保对象4.2万余人。建设敬老院、光荣院、福利院82所。新增养老床位7000张，每千名老年人拥有养老床位数34张，提前一年达到国家目标。“救急难”试点取得初步成效。章贡区“智慧社区”系统试点运行。

各项社会事业全面进步。乡镇公办幼儿园建设竣工135所，在建113所，新增公办学前教育学位7万多个，农村入园难问题得到有效缓解。义务教育学校标准化建设竣工757所，累计新（改、扩）建校舍283万平方米。赣州被列为国家公共文化服务体系标准化试点城市。“三馆一站”全面免费开放。成功承办第十四届省运会，金牌数和团体总分均列全省第二，创历史新高。重大疾病免费救治和新农合大病保险试点顺利开展。二级以上公立医院门诊、住院次均费用实现“双下降”。食品药品安全状况总体平稳。“送政策、送温暖、送服务”工作深入推进。信访、维稳工作得到加强，公众安全感指数居全省前列。国防动员、民兵预备役建设、人民防空、双拥共建和优抚安置工作不断加强。

按照市委统一部署，深入开展政府系统党的群众路线教育实践活动，严格落实中央八项规定和国务院“约法三章”，“四风”突出问题得到有效整治，政府作风和效能建设明显加强。全市“三公”经费支出下降16.2%，市本级下降22.1%。基本完成市县政府机构改革和事业单位分类改革。市政府应急指挥平台和数字化统一办公平台建成运行。加大力度简政放权，公布行政审批事项目录清单，市本级行政审批事项精简41.9%，平均办结时限缩短45%。自觉接受市人大法律监督和市政协民主监督，办理建议、提案576件，交办率、办结率、满意和基本满意率均达100%。规范性文件和重大行政决策合法性审查、重大决策社会稳定风险评估等制度更加健全。廉能风险防控取得实效，政府廉政建设不断加强。

各位代表，回顾过去的一年，困难比预料的多，压力比预想的大，付出的艰辛非比寻常，成绩来之不易！这是党中央、国务院深切关怀，省委、省政府和市委正确领导的结果，是全市上下同心协力、拼搏进取的结果。在此，我代表市人民政府，向全市广大工人、农民、知识分子、干部和历任老领导、老同志，向各民主党派、工商联、无党派和社会各界人士，向驻市人民解放军、武警官兵和公安干警，向国家部委对口支援我市的全体干部，向所有关心支持赣州发展的同志们、朋友们，表示衷心的感谢和崇高的敬意！

在充分肯定成绩的同时，我们也清醒地看到存在的困难和问题，主要是：经济总量不大，人均水平不高，与全国同步全面建成小康社会差距和压力较大；基础设施仍然滞后，要素制约仍然突出，一些体制机制障碍仍然存在；创新驱动能力不强，战略性新兴产业规模偏小，转型升级任务艰巨；社会事业欠账多，扶贫攻坚任务重，改善民生压力大；政府作风建设成果有待巩固，行政效能有待提高，法治政府建设有待加强。对此，我们将直面以待，不回避、不掩饰、不懈怠，努力通过全面深化改革加快解决。

二、2015年主要任务

2015年是全面深化改革的关键之年、全面推进依法治国的开局之年、全面完成“十二五”规划的收官之年，也是为实现振兴发展“五年上台阶、八年大跨越”目标奠定更加坚实基础的重要一年。世界经济仍处在深度调整期，总体延续缓慢复苏态势。我国经济发展进入新常态，经济增速从高速转向中高速，增长结构从中低端转向中高端，增长动力由传统增长点转向新增长点。我们将紧紧扭住振兴发展这一战略主线，适应新常态、构筑新优势、创造新业绩，不断开拓振兴发展新境界。

今年政府工作的总体要求是：全面落实党的十八大、十八届三中、四中全会和中央经济工作会议精神，以邓小平理论、“三个代表”重要思想、科学发展观为指导，深入贯彻习近平总书记系列重要讲话精神，按照省委“发展升级、小康提速、绿色崛起、实干兴赣”十六字方针，以稳中求进为工作总基调，坚持实施《若干意见》为总抓手，坚持加速发展与注重质量相统一，坚持深化改革与扩大开放相促进，强化创新驱动，强化民生保障，强化法治建设，纵深推进赣南苏区振兴发展，为与全国同步全面建成小康社会打下更加坚实的基础。

全市经济社会发展主要目标是：生产总值增长9.5%，财政总收入和一般公共预算收入分别增长10%，规模以上工业增加值增长12%，固定资产投资增长20%，社会消费品零售总额增长12.5%，外贸出口总额增长7%，实际利用外资增长10%，城乡居民人均可支配收入分别增长10%和11%，人口自然增长率控制在8.02‰以内，居民消费价格总水平涨幅控制在3%左右，城镇登记失业率控制在4.5%以内，节能减排完成省下达的计划任务。

重点做好八个方面的工作：

（一）深入推进《若干意见》贯彻落实

继续在内外兼修上狠下功夫，推进《若干意见》扶持政策深化、实化、项目化，构筑振兴发展新优势。

增进振兴发展政策效应。继续抓好现有政策落实，不断深化对市情的再认识、对《若干意见》的再学习，努力在出台赣州市鼓励类产业目录、行政区划调整、生态补偿试点等方面取得新突破。争取瑞兴于经济振兴试验区、国家旅游扶贫试验区规划获批，抓好现有重大平台建设，扩大实物工作量。健全对口支援上下协调推进机制，落实对口支援部委出台的支持意见，持续推动央企入赣活动，争取国家部委安排或推介更多项目在赣州实施，以更大力度加强对赣州的人才培养。

积极融入国家发展新战略。加强与“一带一路”、长江经济带等国家战略的互联互通。对接昌九一体化，融入长江经济带，对接广东、福建自贸区，融入“21世纪海上丝绸之路”，统筹推进各类交通设施建设和多式联运，打造区域性综合交通枢纽，构筑南接北联、通江达海的区域开放发展新格局。科学编制“十三五”规划，认真开展重大课题研究，积极争取我市更多规划、项目纳入国家战略的支撑内容，列入国家和省的规划盘子。

加快重大基础设施项目建设。加快昌赣客专建设，建成赣龙铁路扩能项目，争取开工赣深客专、吉永泉铁路，做好鹰梅铁路、赣井铁路前期工作。建成南昌至宁都高速公路、寻全高速寻乌至安远段，加快兴国至赣县、广昌至吉安（宁都段）、宁都至定南（赣粤界）和定南联络线高速公路建设，升级改造国省道394公里。推进黄金机场改扩建，抓紧瑞金支线机场前期工作。开工华能瑞金电厂二期工程，做好泉州-赣州、揭阳-梅州-赣州成品油管道前期工作。加快500千伏赣州东（红都）输变电工程建设，实现县县覆盖220千伏电网。

（二）加快推进改革创新

狠抓各项改革措施落地生根，加速释放改革红利，充分激发发展活力，打造改革创新先行区。

深化重点领域改革。抓紧抓实国家批复的34个试点示范事项，争取国家部委更多改革试点布局赣州。深化市县政府机构改革，推进政府权力清单、责任清单、负面清单和网上审批系统等“三单一网”改革，进一步精简审批事项，清理非行政许可审批项目，清理规范行政事业性收费项目。深化农业农村改革，构建市、县、乡三级农村产权流转交易服务体系。做好不动产统一登记工作。完成农村土地承包经营权、集体建设用地使用权、宅基地使用权确权登记颁证工作，扩大农民住房财产权抵押贷款试点范围，探索农村集体经营性建设用地改革试点、农村集体资产股份制改革试点和农村宅基地有序退出试点。顺应转移支付制度改革方向，深化财税制度改革。扩大“营改增”和政府购买公共服务试点范围。健全完善全口径预算体系，全面推进财政预决算信息公开。清理规范各类税收优惠政策，加强政府债务管理。推进金融综合改革创新试验区建设。深化国资国企改革，开展国企混合所有制试点。推进社会信用体系建设，启动公共信用信息平台建设。开展机关事业单位工作人员养老保险制度改革。推动教育综合改革、公立医院改革、社会治理创新等改革取得新突破。

充分激发市场活力。进一步放宽市场准入，清理不合理规定，落实扶持政策措施，激发非公有制经济发展活力和创造力。继续深化商事制度改革，减少工商登记前置许可事项，依法推进“先照后证”，加强事中事后监管。大力推广“财园信贷通”“小微信贷通”“财政惠农信贷通”，组建中小微企业服务中心和小微企业融资征信服务平台，支持“专、精、特、新”中小微企业健康发展。稳步推行政府与社会资本合作（PPP）模式，通过特许经营、购买服务、股权合作等方式，鼓励社会资本参与提供公共产品和公共服务并获取合理回报。设立产业投资引导基金，撬动和吸引社会资本参与投资，通过政府和社会、民间资金协同发力，促进大众创业、万众创新。

鼓励支持科技创新。加快科技创新体系建设，强化企业创新主体地位，引导创新要素向企业集聚。充分发挥国家离子型稀土资源高效开发利用工程技术研究中心、脐橙工程技术研究中心等国家级科技创新平台带动支撑作用，充分发挥大专院校和科研院所人才支撑作用，加速科技要素集聚和企业协同创新，推进产学研用一体化，促进创新成果运用转化。发挥科技创业投资引导基金作用，支持科技型企业开展技术创新融资，推进科技与金融深度结合。建立健全科技型人才引进、培养、使用和管理机制，为科技创新提供人才保障。

以信息化引领创新发展。大力发展以互联网为核心的信息经济，促进虚拟经济与实体经济融合互动、信息技术与传统产业融合创新。加快“宽带中国·光网城市”、“宽带乡村”等信息化基础设施建设，大力发展移动互联网，推进智慧城市、智能交通、数字城管建设，推广章贡区“智慧社区”建设做法，提升信息化应用水平。推动生产基地、供应基地与互联网高效对接，依托互联网平台发展新型商业模式。积极引进云计算、大数据企业。鼓励互联网金融创新发展。启动物联网工程建设，争取成为“中国智能物流骨干网”联接四省的区域性物流枢纽。抓好国家电子商务示范城市创建，搭建特色产业电子商务平台，开展电子商务进农村试点，推动特色农产品、优质旅游资源等网络营销。

（三）着力推进产业转型升级

强化龙头企业和重大项目带动，集聚要素培育千亿元产业和百亿元企业，壮大提升特色优势产业集群。

改造提升传统产业。支持企业实施技术改造和兼并重组，淘汰落后产能。深入推进质量强市，积极开展名牌培育工作。加快有色、机械制造、纺织服装、建材、食品等传统产业高端化、低碳化、智能化。充分发挥家具产品质量监督检验、烘干、喷涂、电商中心等公共服务平台作用，推动家具产业向千亿元产业迈进。着力打造服务业发展示范基地，创建省级现代服务业集聚示范区。加快建设现代商贸流通体系，实施赣州市城市共同配送试点项目，推动物流业标准化、专业化、集约化发展。加大旅游资源整合力度，做大做强旅游投资集团。实施乡村旅游富民等工程，培育多元旅游业态，促进传统旅游向现代旅游转型升级。积极引进域外银行，争取第三方支付、民间融资登记服务机构、消费类金融等创新试点，培育壮大地方法人金融机构。加快建设多层次资本市场，支持前海股权交易中心赣州办事处创新发展，拓宽企业融资渠道。实施养老健康家政、信息、旅游、住房、绿色、教育文化体育等六大领域消费工程，培育新的消费增长点。

加快培育新兴产业。推动新能源汽车及其配套、生物医药、新材料、新能源、节能环保等战略性新兴产业集聚发展。充分利用国家资源税“从价计征”改革契机，推动稀土、钨产业链向高附加值环节延伸。积极引进建设新能源汽车整车生产项目，大力发展动力电池、驱动电机等关键零部件，完善配套产业，提升新能源汽车产业规模和效益。培育壮大移动通信终端、数字家庭、新型显示、半导体照明、北斗导航等新一代电子信息产业。

发展壮大现代特色高效农业。落实中央一号文件，多措并举增加农民收入。确保粮食总产稳定在280万吨以上。持续打好柑橘黄龙病防控攻坚战，推进赣南脐橙品牌提升、自营出口、Ο2Ο运营，完善脐橙质量标准体系。着力提升烟叶种植、加工、销售等环节规模和效益，加快构建烟草产业全产业链。推广节水减污等新型生猪养殖技术，发展设施蔬菜，推进茶叶品牌整合，提升油茶、花卉苗木、工业原料林等优势特色产业发展水平。强化农产品质量安全监管，提高农业标准化生产水平。深入实施全省“百县百园”工程，抓好现代农业示范园区建设。推进新型职业农民培养和新型农业经营主体培育融合发展，全年培训农民20万人次，发展农民合作社500家、家庭农场300家，新增龙头企业30家。

（四）大力推进新型城镇化

用好新型城镇化这个强大引擎，促进城镇化加速健康发展，切实优化经济发展空间格局。

做大做强市中心城区。健全空间规划体系，推进“多规合一”试点。深入实施赣州都市区总体规划，开展赣州中心城市近期建设规划（2016-2020年）、地下管线综合规划、新能源汽车充（换）电桩专项规划、电力设施专项规划等编制工作。按照“一带三轴六区”城市空间发展总体结构，加快蓉江新城、西城区及香港产业园北区、赣州职教园区、水东组团、南康东山新区建设。继续推进“四桥九路”等重大城建项目建设，建成黄金、武陵、客家大桥。加快推进上犹江引水工程。实施南门口下穿隧道等交通节点改造，优化城市交通。建立地下空间综合管理信息系统，提升城市地下空间开发利用水平。抓好供水供气、地下管网、农贸市场、停车场、小游园、公厕等市政设施和公共服务项目建设，加快第二垃圾处理场和贡江左岸截污干管建设。纵深推进“治脏、治乱、治堵”，建设“干干净净、漂漂亮亮、井然有序、和谐宜居”的城市家园。

加快城乡统筹发展。推进瑞金、龙南两个市域副中心城市与国家级经济技术开发区融合发展。支持各县统筹抓好旧城改造和新区建设，同步推进道路、供水、环保等基础设施向农村延伸拓展。抓好示范镇建设，着力打造一批文化旅游、商贸物流、资源加工、交通枢纽等特色城镇，构建大中小城市和小城镇协调发展格局。推进新一轮新农村建设，启动村庄规划编制，突出镇村联动和中心村建设。开展“清洁田园、清洁家园、清洁水源”行动，完善新型农村社区配套设施，提升城乡基本公共服务均等化水平。深入推进户籍制度改革，加快农业转移人口市民化。

发展壮大县域经济。加强对县域产业的规划和指导。以赣州都市区综合交通体系规划为引领，以高铁建设为纽带，提前谋划昌赣、赣深客专等沿线城镇、区域的发展定位和产业布局。制定实施差异化扶持政策，鼓励引导各县（市、区）主攻1至2个特色产业集群错位发展，夯实县域经济发展的产业支撑。完善县域经济发展考评体系，实行分类考核、差异化考核。深入推进省直管县、扩权强县、扩权强镇改革试点，最大限度为县域经济发展“松绑给力”。

（五）积极推进开放型经济发展

充分发挥政策、区位、资源、平台等优势，坚持“引进来”和“走出去”相结合，不断拓展对外开放广度和深度。

着力招大引强。突出珠三角、长三角及厦漳泉等重点区域，紧盯国内外五百强、中央企业和大型民企等目标企业，重点引进精密机械制造、高端装备制造等产业项目落户。针对重点项目，采取“高频率、多批次、专业化、小分队”的方式，一对一推介洽谈，提升招商引资实效。加强与赣商和各商会的联系，吸引鼓励赣商抱团返乡创业。强化招商引资考核，建立健全招商引资项目常态化调度机制，提高签约项目履约率、开工率、投产率。

深化开放合作。借鉴上海自贸区试点经验，策应福建平潭综合实验区、厦门综合配套改革试验区和福建、广东自贸区建设，加快赣闽、赣粤产业合作区建设，深化与港澳台地区的交流合作。充分发挥外贸促进中心服务功能和外贸专项资金引导作用，鼓励支持企业参与国际竞争，开拓国际市场。

抓好开放平台建设。建成赣州综合保税区一期并封关运行。加快赣州、龙南、瑞金经济技术开发区和赣南承接产业转移示范区建设，争取赣州高新技术产业园区升格为国家级、“三南”承接加工贸易转移示范地授牌。推动赣州进境木材监管区、定南公路口岸作业区常态化运营，加快赣州铁路口岸作业区扩容改造，规划建设进境水果、进境再生原料、进境饲料用粮等监管区。实施园区提升计划，推进省级经济技术开发区及工业园扩区调区，为扩大开放提供有力支撑。

（六）有效推进生态建设和环境保护

贯彻落实江西省生态文明先行示范区建设部署要求，加速构筑我国南方地区重要生态屏障。

加强环境综合治理。深入开展“净空、净水、净土”行动。加强市中心城区PM2.5监测和大气污染防治，加快污染企业搬迁改造，实施“煤改电”“煤改气”工程和重点行业脱硫、脱硝、除尘改造，淘汰黄标车和老旧机动车，确保环境空气质量保持优良。抓好全国水土保持改革试验区建设和矿山地质环境治理。加强农业面源污染防控、土壤污染源头综合整治和重金属污染防治，加大主要河流流域综合整治和陡水湖生态环境保护治理力度，提高城镇污水处理率，确保城镇集中式饮用水水源地水质达标率稳定在100%。抓好重点节能减排项目实施和公共机构节能，全面完成“十二五”节能减排任务。

促进经济生态融合。大力发展生态经济，构建区域生态产业体系。推进生态旅游业、低碳农业、林下经济等绿色产业集群化发展，形成以低碳排放为特征的新经济增长点。加快建设稀有稀土金属、铜铝有色金属循环经济产业园，做大有色金属循环经济产业。抓好国家生态工业示范园区、国家林业科技示范园区建设，创建国家级森林城市。

完善生态文明建设机制。深入推进国家低碳试点城市建设，建立完善低碳发展法规保障体系、政策支撑体系、技术创新体系和激励约束机制，形成具有赣州特色的低碳城市发展模式。开展领导干部自然资源资产离任审计试点。用好碳排放权交易平台，推进碳汇造林和碳减排指标有偿交易。探索建立资源有偿使用和生态补偿制度，争取《江西东江源生态保护补偿规划》尽早获国家批复。

（七）全面推进民生社会事业发展

坚持人民政府为人民，顺应民心关切，切实解决好群众最关心、最直接、最现实的利益问题，促进发展成果更多、更公平惠及全市人民。

办好民生实事。筹集资金300亿元以上保障民生支出，办好40件民生实事，并完成省下达50件民生实事。全面完成农村危旧土坯房改造任务，实施农村安全饮水提质增效工程，持续推进农村低电压治理，做好农村义务教育薄弱学校改造工作。新建改造农村公路3200公里，改造危桥100座。加大保障性安居工程建设力度，新建公租房和改造各类棚户区2.1万套。实施高校毕业生就业促进计划、创业引领计划、农民工职业技能提升计划，大力扶持就业创业。扩大社会保险覆盖面，完善城乡养老保险制度衔接政策和被征地农民养老保险政策，推进医疗保险城乡统筹。进一步完善城乡低保制度，扩大农村低保覆盖面。深入开展“救急难”试点。

实施精准扶贫。市、县两级财政各筹资10亿元，设立20亿元扶贫救助专项资金，为扶贫对象从事生产经营活动提供贷款担保。推进区域开发与精准扶贫相结合，坚持因地制宜、因户施策，通过产业扶贫、移民扶贫、教育扶贫、就业扶贫、保障扶贫、金融扶贫等多措并举，一对一解决突出问题。实施扶贫移民搬迁2.5万人，力争全年脱贫20万人。

发展各项事业。抓好部省共建赣州教育改革发展试验区建设，基本解决小学、初中寄宿生住宿问题，完成乡镇公办中心幼儿园、农村义务教育学校标准化建设。建成并投入使用市人民医院新院，加快市儿童医院建设。继续开展“看病难、看病贵”问题专项整治，推进重大疾病免费救治和新农合大病保险，加强重点传染病防控。推进综合福利院、光荣院、敬老院建设，大力发展民办养老服务机构。完善城乡居家养老和社区养老服务网络，统筹利用各种资源，促进养老服务与医疗、家政、保险、教育、健身、旅游等互动发展。加快建设红色文化传承创新区、国家公共文化服务体系示范区、国家级客家文化（赣南）生态保护实验区。完成天竺山烈士陵园改造工程。加强社区服务中心（站）建设，完善社区服务和治理结构。加快公共体育设施建设，办好第四届市运会。优化整合计划生育利益导向政策，促进人口长期均衡发展。

创新社会治理。全面推进法治赣州建设，坚持用法治思维和法治方式深化改革、推动发展、化解矛盾、维护稳定，着力保障社会公正、促进社会诚信、维护社会秩序。扎实做好“送政策、送温暖、送服务”工作。完善矛盾纠纷排查调处、诉访分离、涉稳信息预警和就地化解机制，推进信访工作改革，推动群众合理合法诉求依法、及时、就地解决。做好流动人口和重点特殊人群服务管理。加强社会组织和互联网管理。健全以安全生产、食品药品安全、防灾减灾救灾、应急管理、社会治安防控等为基本内容的公共安全体系，依法严厉打击各类违法犯罪活动，不断提高人民群众安全感和满意度。

（八）扎实推进政府自身建设

通过改革促进观念、职能、作风转变，推动政府工作在法治轨道上提速提质提效，不断提升政府服务和治理能力。

加快建设法治政府。健全依法决策机制，完善行政决策规则和程序。积极推行政府法律顾问制度，严格重大行政决策合法性审查。深化行政执法体制改革，开展综合执法试点，严格规范公正文明执法。开展市本级行政复议委员会试点。推进政府决策公开、执行公开、管理公开、服务公开、结果公开。做好人大代表建议和政协提案办理工作。自觉接受人大法律监督和政协民主监督，主动接受司法监督、舆论监督和社会监督，强化监察、审计等内部监督，形成科学有效的行政权力运行制约和监督体系。把政务履约和守诺服务纳入政府绩效评价体系，以诚信办事、诚信服务取信于民。

坚持不懈改进作风。巩固提升群众路线教育实践活动成果，坚决贯彻中央八项规定，严格执行国务院“约法三章”，持之以恒整治“四风”。严格控制“三公”消费和一般性开支，完成行政机关及参公事业单位公务用车制度改革。严格落实党风廉政建设责任制，认真履行“一岗双责”，推进廉政风险防控管理。加大腐败案件查处力度，坚决纠正部门和行业不正之风，大力营造风清气正的政务环境。

狠抓各项工作落实。着力提升能力抓落实，不断加强对新政策、新法规、新知识的学习，提高政策领悟力和实践操作力；着力改进方法抓落实，始终把握正确方向，突出重点难点，深入实际认真研究新情况、解决新问题、探索新路径；着力完善机制抓落实，建立健全督促检查、考核奖惩、责任追究等制度，做到“年初建账、年中查账、年底交账”；着力强化担当抓落实，敢于直面矛盾、勇于担当责任、善于攻坚克难，确保各项决策落到实处，确保全年目标任务圆满完成。

各位代表！实干铸就辉煌，奋斗成就伟业。让我们更加紧密地团结在以习近平同志为总书记的党中央周围，在市委的坚强领导下，保持定力，砥砺前行，改革创新，开拓进取，奋力谱写全面深化改革、全面振兴发展、全面建成小康新篇章！
